# Prevalence of Animal Fasciolosis and Specification of *Fasciola* spp. Isolated from Sheep, Goats and Cattle by Molecular Method: Hamadan Province, West of Iran

**Published:** 2018

**Authors:** Kobra PIRI, Massoud SAIDIJAM, Amirhossein MAGHSOOD, Mohammad MATINI, Mohammad FALLAH

**Affiliations:** 1. Dept. of Parasitology and Mycology, School of Medicine, Hamadan University of Medical Sciences, Hamadan, Iran; 2. Dept. of Molecular Medicine and Genetics, School of Medicine, Hamadan University of Medical Sciences, Hamadan, Iran

**Keywords:** Prevalence, *Fasciola*, PCR-RFLP, Iran

## Abstract

**Background:**

Fascioliasis is a common disease among humans and animals. Having global distribution, disease is developed by hepatic trematodes, *Fasciola hepatica* and *F. gigantica*. The main objective of this research was determining the prevalence of *Fasciola* species in Hamadan livestock and identifying those using PCR-RFLP.

**Methods:**

Overall, 13607 livestock livers in the slaughterhouse of Hamadan, west of Iran including 10846 sheep, 995 cattle and 1766 goats were examined in 2015. In addition, 75 *Fasciola* (41 worms from sheep, 22 worms from goats and 12 worms from cattle) were examined by PCR-RFLP method.

**Results:**

Totally, 100 livers were infected to *Fasciola* species (total prevalence: 0.74%; sheep 0.5%, goats 1.4%, cattle 1.5%). In the molecular results, prevalence of *F. hepatica* was higher than (92.5% in the sheep) *F. gigantic*, and in the cattle 91.5% and in the goats was 54.5% *F. hepatica*. Genotyping identified species confirmed intermediated types as *F. hepatica*.

**Conclusion:**

Prevalence of *Fasciola* in this province is not so high. Intermediate types identified by PCR-RFLP method determined as *F. hepatica* by nucleotide sequencing. Because of morphological differences and interspecies variety, the accurate identification of *Fasciola* species needs using nucleotide sequencing.

## Introduction

Fasciolosis is a common disease among animals and humans (1). In human cases, the parasite can lead to inflammation in liver and bile ducts (2). In livestock, it leads to many economic losses including reduction in milk, meat, wool (2, 3). In recent years, disease control importance has become more prominent due to the changing in its epidemiologic pattern (2). There are some intermediate species in addition to two common known species (*F. hepatica* and *F. gigantica*) (4–6). The morphological characteristics of adult worms are affected by different factors such as parasite’s age, type of the host, intensity of infection and the used method for species identification; therefore, the traditional methods based on the morphological characteristics are unable to specification accurately(2, 7). On the other hand, these species may have cross-fertilization and create new hybrid types that may not differentiable using morphological methods (8, 9). Therefore, considering to economic and human health importance of fasciolosis, the accurate identification of causing species seems necessary in order to prevent this disease and adopting control methods properly.

Usually, *Fasciola* species are identified based on morphological criteria such as width and length of cephalic cone, length of area between back of testicles and tail of the worm, length to width ratio of adult worm etc. (10, 11). However, considering to intermediate and hybrid forms, these criterions may not be accurate and reliable.

Therefore, the main objective of this study was determining the prevalence of *Fasciola* species in Hamadan livestock and identifying species using PCR-RFLP method and nucleotides sequencing.

## Methods

### Parasite

Overall, 13607 livestock livers including 10846 sheep, 995 cattle and 1766 goats in the Hamadan slaughterhouse were examined during 2015. Totally, 100 livers were infected by *Fasciola spp*. In addition, 75 *Fasciola* worms (41 from sheep, 22 from goats and 12 from cattle) were examined by, PCR-RFLP. Based on the morphology of different species of *Fasciola* in each animal, these species were put in containers with 70% alcohol separately, after collecting and washing in saline and were kept in −20° C after recording the worm’s characteristics.

### DNA extraction

Phenol-chloroform method carried out in order to genomic DNA extraction. Briefly, the proximal part of the worm (the area between oral sucker and ventral sucker) was cut using sterile scalpel on a slide and then it was put to 1.5 ml micro-tubes containing 500 ml lysis buffer solution and 10 ml proteinase K with 20 mg/ml concentration. After a 5-sec vortex, micro-tubes were put in an incubator in 37° C temperature overnight. After centrifuging for 10 min with 100 ml phenol-chloroform, the supernatant was separated and twofold its volume was added the cold absolute ethanol. After putting in −20°C overnight, the fluid was re-centrifuged. About 50 ml of sterile distilled water was added to micro-tubes and were held in 4 °C until performing the molecular test.

### Molecular method

In molecular method, the used gene was ITS1 with ITS1-F primer and primer sequence of 5-TTGCGCTGATTACGTCCCTG-3 and ITS1-R primer with primer sequence of 5-TTGGCTGCGCTCTTCATCGAC-3(12).

### PCR method

The primers used in this study were according to protocol reported previously(12). In order to amplification of the DNA samples, Master Mix prepared using PCR buffer (10x) (2.5 ml), dNTP 10 mM (0.5 ml), MgCl2 50 Mm (1 ml), reverse primer (10 pmol) (0.5 ml), forward primer (10 pmol) (0.5 ml), Taq DNA polymerase (2.5 U/ml) (0.5 ml) (all materials from Cinnagen Company, Iran), DNA (3 ml) and DD water (16.5 ml). In order to perform PCR, the thermo-cycler program was used with three steps including denaturing (94°C for 3 min), annealing [(94°C for 90 sec, 55°C for 90 sec and 72°C for 2 min and 30 cycles]; and extension step: (72° C for10 min). The PCR products were loaded on 1% agarose gel subjected to electrophoresis. PCR product was used to RFLP in case of having sharp band.

### PCR-RFLP analysis

This method was performed on PCR products in ITS1 genomic area in order to identify *Fasciola* species accurately. Then, Master Mix was prepared in 30 ml final volume. After preparing Master Mix in 0.5 ml micro-tube, it was kept in a 37°C incubator for 3.5 h; the pieces resulted from *Rsa1*enzyme cut were visualized by UV illumination on 3% agarose gel.

### Sequencing method

In order to identify nucleotides’ sequence in isolated *Fasciola* after PCR-RFLP analysis, 7 samples (4 cases identified as intermediate type morphologically, 2 *F. hepatica* and 1 *F. gigantica*) were chosen. Master Mix was prepared in 50 ml volume and PCR was performed on these samples again. After NANO-drop, PCR products of the samples was separately poured in 1.5 ml micro-tubes and tubes’ opening covered by para-film. In addition, 10 ml forward primer and 10 ml reverse primer (5 pmol each) added to each micro-tube and covered by para-film. Finally, in order to sequencing, the samples were sent to Bioneer Co, South Korea. Sequencing of 680 bp genes of 18s rRNA was aligned with sequencing of nucleotides in genes of GenBank (NCBI) using BLAST (Basic Local Alignment Search Tool) software.

## Results

Amongst all studied livestock, 100 livers (sheep 60, goat 25 and cattle 15) were identified infected to *Fasciola* species (overall prevalence 0.74%; sheep 0.5%, goats 1.4%, cattle 1.5%).There was no significant difference between infection rate and sex or age the slaughtered animals. Intensity of infection was higher in cattle (22.07±26.13) than goats (17.42±15.22) and sheep (7.62±11.28) (*P*<0.018). There was not significant difference between intensity of infection and sex but, the younger animals had intensity of infection higher than olds (13.35±12.76 vs 11.98±16.88) (*P*<0.05).

Table 1 shows prevalence of *Fasciola* species in the livestock using PCR-RFLP. According to the molecular results, prevalence of *F. hepatica* in all three types of livestock was higher than prevalence of *F. gigantica* and suspected intermediate type.

**Table 1:** Number and Prevalence of *Fasciola* species in the slaughterhouse of Hamadan in terms of type of livestock using PCR-RFLP

***Livestock***	***F. hepatica N(%)***	***F. gigantica N(%)***	***Suspected to intermediate typeN(%)***	***Total prevalence N(%)***
Cattle	11 (91.7)	1 (8.3)	0	12 (100)
Sheep	38 (92.68)	0 (0)	3 (7.32)	41 (100)
Goat	12 54.5)	9 (40.9)	1 (4.5)	22 (100)
Total	61 (81.33)	10 (13.33)	4 (5.33)	75 (100)

**Fig. 1:**
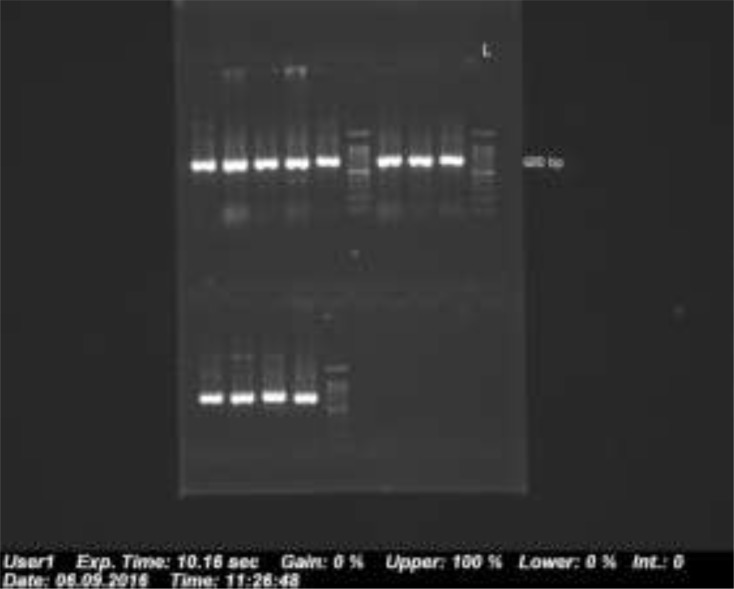
The PCR product of 680 bp piece confirming type of *Fasciola*, the right column Ladder (100 bp)

**Fig. 2:**
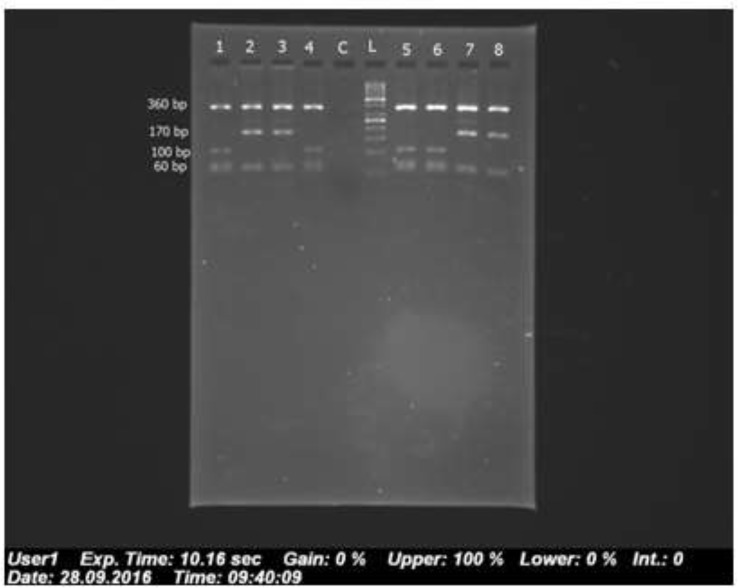
RFLP pattern of PCR products of *F. hepatica* and *F. gigantica* using *Rsa1* enzyme. Samples’ number, 1 to 4 are positive control (1 and 4 are *F. hepatica*, 2 and 3 are *F. gigantica*), C is negative control, L is Ladder (50 bp), 5 and 6 are *F. hepatica*, and 7 and 8 are *F. gigantica*

**Fig. 3:**
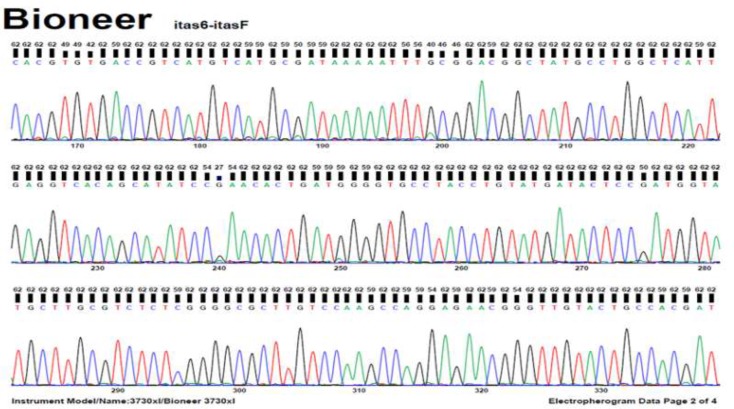
Florescence Graph18S rRNA *Fasciola hepatica*, 680 bp

**Fig. 4:**
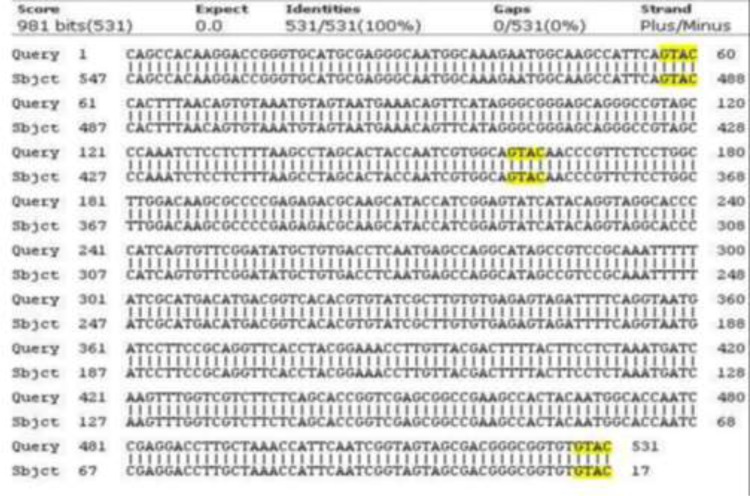
Conformity of *F. hepatica* 18S rRNA with the location of *Rsa1* enzyme cutting, 680 bp

**Fig. 5:**
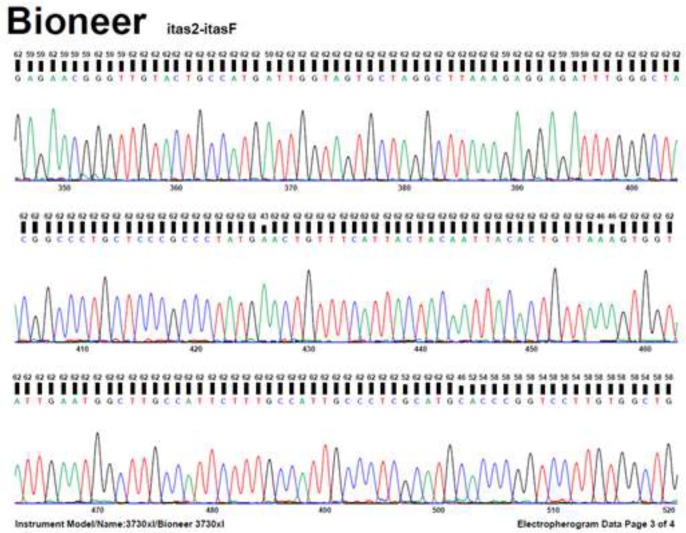
Florescence Graph 18S rRNA *F. gigantica*, 680 bp

**Fig. 6:**
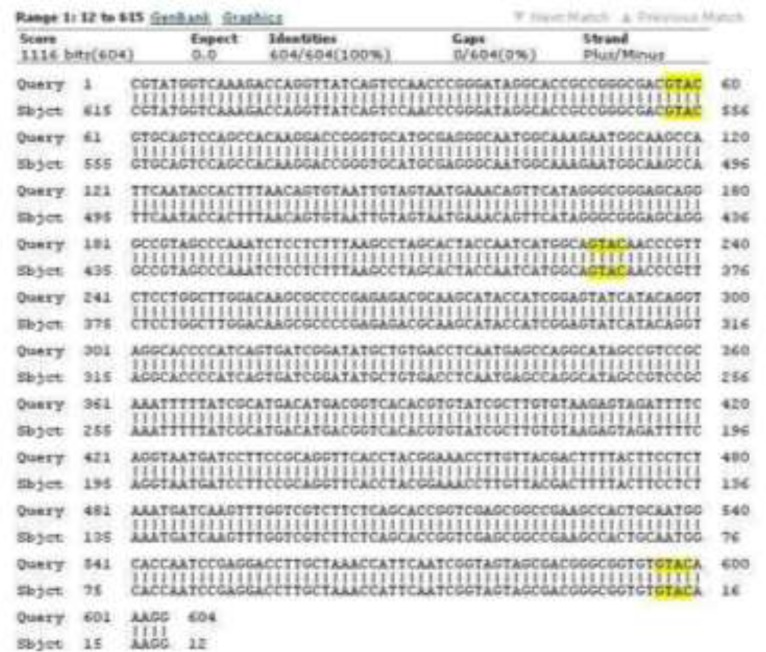
Conformity of *F. gigantic* 18S rRNA with the location of *Rsa1* enzyme cutting, 680 bp

The Fig. 1 shows a 680 bp band that confirmed the *Fasciola* genus. Fig. 2 shows RFLP pattern of PCR products of *F. hepatica* and *F. gigantica* using *Rsa1* enzyme. Fig. 3 is florescence graph 18S rRNA *F.hepatica*, 680 bp. Fig. 4 is conformity of *F. hepatica* 18S rRNA with the location of *Rsa1* enzyme cutting, 680 bp. Fig. 5 shows florescence graph 18S rRNA *F. gigantica*, 680 bp. Fig. 6 shows conformity of *F. gigantica* 18S rRNA with the location of *Rsa1* enzyme cutting, 680 bp.

## Discussion

In Iran, two species of *F. hepatica* and *F. gigantica* has a particular life cycle in different geographical regions (7). Implementation of the proper policies for prevention and control the disease in each region requires accurate identification of the parasite life cycle and its dominant species. Considering the differences in *Fasciola* species and its epidemiologic pattern, careful specifying the parasite is necessary in order to proper implementation of any control program in human and animal fascioliasis. In addition, differentiation of *Fasciola* species is vital due to existence of intermediate and hybrid forms (1, 2). Since morphological indices and criteria and routine differential methods of *Fasciola* species are not accurate and reliable (13–15), molecular methods which have high sensitivity and accuracy in specification of parasite is used in this study (8).

Based on DNA analysis, molecular markers are used for genetic description and parasite identification(16,17). Using the restriction enzymes are a simple and powerful method for parasite specification based on differences in their genomes. This method is used to differentiate *Fasciola* species based on endonuclease effects of ITS region in these parasites (18, 19). In some studies, PCR-RFLP is used to identify *Fasciola* using 28s rRNA, 18s rRNA, ITS1 and ITS2 (15, 20, 21).

According to a report from Tabriz, Iran, using PCR-RFLP and sequences of ITS1, 5.8S and ITS2 areas of ITS region, all the 90 trematode of *Fasciola* identified as *F. hepatica* (70 worms) and *F. gigantic*a (20 worms)(22). Nucleotide sequencing of *Fasciola* isolated from sheep and human (by sequencing ITS-1 and ITS2 region) indicated that 99%–100% cases of sequencing ITS-1 and ITS2 for *Fasciola* isolated from different regions and different hosts were similar (23). Using PCR-RFLP method from 48 isolated *Fasciola*, 4 isolates from goats (100%) were *F. hepatica*, 27 isolates (96%) and 1 isolate (4%) from cattle were *F. hepatica* and *F. gigantica*, respectively (24). Among 80 isolates, 50 isolates were *F. hepatica* and 30 isolates were *F. gigantica* morphologically; no hybrid pattern was found and there was no difference between morphological method and PCR-restriction enzyme for specification (21). In addition, using PCR-RFLP method and nucleotides sequencing (25), the genotype of *Fasciola* intermediate type in examined samples identified as *F. hepatica*.

Restriction enzymes in 28s DNA, named *Ava* II and *Dra* II indicated the differences between two species of *F. hepatica* and *F. gigantica* correctly (26). In sequencing analysis ITS1 and *nad*1 in genetic examination, 11 out of 17 cases of Fasciolosis were due to *F. hepatica*, 4 cases were *F. gigantica* and 2 cases were mixed infections of both *Fasciola* species (27). In Japan, *Fasciola* species were identified using PCR-RFLP and *Rsa1* enzyme on ITS1 gene, i.e. out of 25 applied isolated samples, 10 cases were *F. hepatica*, 9 cases were *F. gigantica* and 6 cases identified as intermediate type.

Sequencing ITS1 and ITS2 fragments is a proper genetic marker to identify genotype, interspecies variety and phylogenetic characteristics (28). Using ribosomal sequencing ITS1 and ITS2, only 0.86% (5 cases) were *F. gigantica* based on morphological characteristics, while 100% of cases were *F. hepatica* based on sequencing ITS1 and ITS2. Among 10 registered sequences in Iran’s GenBank, 62.6% were *F. hepatica*, 24.3% were *F. gigantic*a and 13.1% were registered as *Fasciola* spp. Since molecular method based on DNA analysis is more trustable to identify species of *Fasciola* in comparison to other methods, therefore, DNA markers required to identify species of *Fasciola* include COI, ITS2, 5.8s, ITS1 and ND1 (29).

## Conclusion

*F. hepatica* is the dominant species in Hamadan. Some intermediate types were identified by PCR-RFLP method while all of them were identified as *F. hepatica* after sequencing of nucleotides. Identification of species based on PCR-RFLP method may not be accurate without nucleotides sequencing. Meanwhile, PCR-RFLP is a simple, quick, cheap and available tool that is able to differentiate *F. hepatica*. and *F. gigantica* species. However, the best and most definitive method is nucleotide sequencing of this parasite.
